# Mechanisms of Peripheral and Central Sensitization in Osteoarthritis Pain

**DOI:** 10.7759/cureus.35331

**Published:** 2023-02-22

**Authors:** Yoshihisa Ohashi, Kentaro Uchida, Kensuke Fukushima, Gen Inoue, Masashi Takaso

**Affiliations:** 1 Department of Orthopaedic Surgery, Kitasato University School of Medicine, Sagamihara, JPN

**Keywords:** nerve growth factor, inflammatory cytokines, pain, central sensitization, peripheral sensitization, osteoarthritis

## Abstract

Pain, the primary symptom of osteoarthritis (OA), reduces both the quality and quantity of life for patients. The pathophysiology of OA pain is complex and often difficult to explain solely by radiological structural changes. One reason for this discrepancy is pain sensitization (peripheral sensitization [PS] and central sensitization [CS]) in OA. Thus, an understanding of pain sensitization is important when considering treatment strategies and development for OA pain. In recent years, pro-inflammatory cytokines, nerve growth factors (NGFs), and serotonin have been identified as causative agents that induce peripheral and central sensitization and are becoming therapeutic targets for OA pain. However, the characteristics of the clinical manifestations of pain sensitization elicited by these molecules remain unclear, and it is not well understood who among OA patients should receive the therapeutic intervention. Thus, this review summarizes evidence on the pathophysiology of peripheral and central sensitization in OA pain and the clinical features and treatment options for this condition. While the majority of the literature supports the existence of pain sensitization in chronic OA pain, clinical identification and treatment of pain sensitization in OA are still in their infancy, and future studies with good methodological quality are needed.

## Introduction and background

Osteoarthritis (OA), the most common form of arthritis, is a painful chronic disease of the synovial joints. Chronic pain and its related symptoms in OA reduce both the quantity and quality of life [1,2]. Understanding OA pain is hindered by the fact that it can be intense or chronic, regardless of the degree of structural change. Indeed, several studies report that the association between radiographic structural changes and pain levels in OA is poor [3,4]. OA pain is subjective, involving both peripheral and central neural mechanisms, which are modulated not only by a wide range of neurochemical factors but additionally by environmental, psychological, and genetic factors [5-7]. Nevertheless, the mechanisms of OA pain are not well understood.

Pain sensitization is considered a key process in chronic pain conditions that are characterized by exaggerated responses to innocuous or only mildly noxious stimuli (hyperalgesia and allodynia) [8]. Two types of sensitization (peripheral sensitization [PS] and central sensitization [CS]) have been reported to affect chronicity and treatment resistance in OA pain [4,5,8-10]. PS is described as the hyperexcitability of peripheral nociceptors and is considered largely due to the effects of neurotrophins and pro-inflammatory molecules in promoting nociceptor depolarization [11,12]. CS by comparison, results from a continuous nociceptive input that occurs as hyperexcitability of wide dynamic range neurons in the dorsal horn (DH) [13]. More understanding of the pathophysiology of pain sensitization in OA may aid in the development of therapies that are better targeted at the direct mechanisms of pain.

Recent evidence suggests that pro-inflammatory cytokines, nerve growth factor (NGF), and serotonin are therapeutic targets for OA pain with PS and CS. This review aims to describe in detail the role of these factors in the mechanisms of pain sensitization, from PS to CS in OA.

## Review

Peripheral sensitization in OA

**Figure 1:**
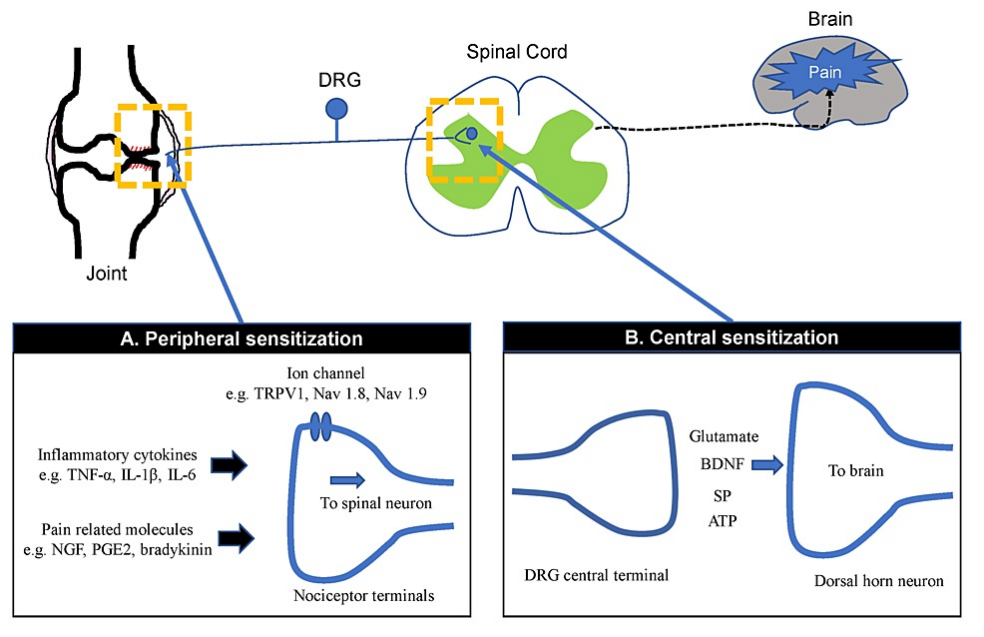
Peripheral sensitization and central sensitization Mediators (inflammatory cytokines, pain-related molecules) released form joint tissues activate peripheral nerve terminals of nociceptor neurons (peripheral sensitization). Persistent pain or inflammation causes activation and repetitive firing in afferent C-fiber nociceptors, which triggers the release of neurotransmitter in the synapse of the dorsal horn (central sensitization). Glutamate, ATP, substance P (SP), and cytokines from DRG central terminals mediate neurotransmission to second-order postsynaptic neurons that relay signals to the brain.

Joint nociceptors are normally inactive but become active during arthritis due to cartilage damage or synovitis. These act to intensify joint pain [14]. In affected synovial tissues, the nociceptive system enters a state of hyperexcitability and can be activated by what are otherwise normal or usually innocuous or mild irritations [15]. Nociceptors in intra-articular tissues are known to be sensitized in electro-physiological studies in OA models in rats and guinea pigs [16,17]. Increased afferent nerve firing rate was observed in a monosodium iodoacetate (MIA)-induced OA model in rats [16]. Afferent nerve firing rate increased with aging in a guinea pig model of spontaneous OA [17]. Also, the mechanical threshold required to activate the afferent nerve fibers was significantly higher in aged guinea pigs compared to younger animals [17]. Inflammation-associated molecules, such as prostaglandins, bradykinin, tumor necrosis factor (TNF)-α, interleukin (IL)-1β, IL-6, damage-associated molecular patterns (DAMPs) are thought to ligate to sensory nerve fibers via transient receptor potential (TRP) channels and sodium channels. This translates into a lower excitation threshold on high-threshold neurons, making joint nociceptors more likely to fire in response to painful stimuli, both noxious and non-noxious [18]. The signals then course via ascending pathways to high central nervous system (CNS) centers and are there interpreted as pain and assigned affective qualities [19]. An overview of the signaling pathways of PS in OA is indicated in Figure 1.

Central sensitization in OA

CS is defined as the elevated responsiveness of nociceptive neurons in the CNS to normal or subthreshold afferent inputs as a result of CNS plasticity [20]. An increase in spontaneous neuronal activity causes pain hypersensitivity by lowering activation thresholds and expanding the receptive field [9]. Pain hypersensitivity includes both hyperalgesia - an increased sensitivity to noxious stimuli- and allodynia - pain as a response to normally innocuous stimuli [21-23]. The mechanism of CS includes excessive nociceptive ascending (sensory) signaling and insufficient inhibitory descending signaling. This facilitation is maintained by peripheral nociceptive input arising from the OA joint itself [24]. DH of the spinal cord is where ascending pathways arise, where they synapse with interneurons or projection neurons that have synapsed with primary afferents. A pain signal is transmitted through these ascending pathways to the hypothalamus, thalamus, brainstem, amygdala, and prefrontal cortex [25]. An overview of the signaling pathways of CS in OA is indicated in Figure 1.

Molecules involved in PS and CS in OA

Pro-inflammatory Cytokines

TNF-α, IL-1β, and IL-6 are potent pro-inflammatory cytokines exerting pleiotropic effects on various cell types and play a critical role in the pathogenesis of chronic inflammatory diseases, such as OA and rheumatoid arthritis (RA). These molecules are released into the joint, and synovial inflammation is associated with pain in OA [26,27]. These cytokines facilitate the firing of joint nociceptors, leading to nociception and the initiation of OA pain [12,28-30]. In a study of rat models, intraarticular TNF-α injection resulted in persistent sensitization of nociceptive Aδ- and C-fibers, which lead to hyperalgesia and mechanical allodynia [12]. It has been suggested that IL‐1β and IL-6 also activate or sensitize nociceptors [29,30]. Furthermore, in animal models of chronic inflammation, primary afferents in the DRG and post-nodal sympathetic fibers were reported to exhibit a neuropathy-like phenotype, with increased sprouting to the affected area and to the DRG itself [31]. Lee et al. revealed that elevated serum IL-6 levels are associated with low-pressure pain thresholds (PPTs) taken at sites remote to the affected joint and high suprathreshold heat pain ratings [32]. Leung et al. reported that concentrations of TNF-α, IL-6, and IL-8 are associated with pain on movement, with only TNF-α being involved in the exacerbation of the pain at rest, which is characteristic of sensitized pain in the synovial fluid of knee OA (KOA) [33]. Further, it is known that these pro-inflammatory cytokines are primarily expressed by synovial monocytes and macrophages in OA joints [34-36]. Further, CD14-positive macrophages regulate NGF via pro-inflammatory cytokine production in the synovial membrane of KOA [34-36]. Synovial CD163 mRNA expression is positively correlated with pain at rest, while CD163+CD14 low macrophages expressing TNF-α might be a major contributor to hip OA (HOA) pain [34]. Considering the evidence, elevated synovial fluid and serum levels of pro-inflammatory cytokines in OA patients might directly trigger PS and contribute to CS.

Nerve Growth Factor

The pronociceptive functions necessarily involved in the pathogenesis of pain include PS and CS, and enhanced local neuronal sprouting at sites of inflammation, within the dorsal root ganglion (DRG), and possibly also within the DH [25]. NGF is associated with these functions. NGF is the founding member of the neurotrophin family of growth factors, which are responsible for the survival, growth, and developmental plasticity of neurons in the peripheral and CNS in vertebrates [37,38]. NGF is produced by chondrocytes, synovial macrophages, and fibroblasts in the osteoarthritic joint [35,39,40]. NGF production was stimulated by transforming growth factor (TGF)-β in osteoarthritic chondrocytes [39]. Synovial fibroblast had higher NGF production ability compared to macrophages following TNF-α stimulation [35,40]. CD14high positive cells had higher NGF expression compared to CD14low cells in HOA [41]. It binds tropomyosin-related kinase A (TrkA), which is expressed in a range of sensory and sympathetic fibers and regulates their survival [42]. TrkA-positive cells account for about 40% of neurons in the DRG. They include thin myelinated Aδ fibers and peptidergic unmyelinated C fibers, both of which innervate multiple tissues [42,43]. The binding of NGF to TrkA on the peripheral terminals of nociceptors and the surface of immune cells may directly play a role in acute PS [42]. NGF/TrkA complex leads to signaling that upregulates the local expression and activation of pronociceptive channels/receptors (Na/Ca/K channels, bradykinin receptors, cation channels, and acid-sensing ion channels) [44-47]. Bradykinin B2 receptor expression was elevated by NGF in mouse DRG culture [44]. The calcium current density increased in cultured embryonic basal forebrain neurons following NGF treatment [48]. NGF directly enhances acid-sensing ion channel 3 encoding genes in DRG neurons [47]. This triggers the sensitization of the nociceptor, resulting in a condition of PS. NGF may contribute indirectly to CS through its downstream influence on transcription. The NGF/TrkA complex is transported retrogradely to neuronal cell bodies in the DRG. The NGF/TrkA signal in turn drives the synthesis of pronociceptive components (brain-derived neurotrophic factor [BDNF], calcitonin gene-related peptide [CGRP], and substance P [SP]) [48-51]. BDNF activates spinal microglia and contributes to the induction and maintenance of the CS [52]. SP and CGRP are released from the peripheral endings of sensory neurons, which contribute to the development of neurogenic inflammation, while SP and CGRP are released from the central termini of sensory neurons, which contribute to enhanced nociception and the buildup of CS [50]. Neuronal sensitization mediated by NGF/TrkA increases nociceptive signaling through the DH and supraspinal structures [42]. The overall effect is the condition of CS.

The release of NGF during cartilage degradation, bone remodeling, and synovial inflammation appears to play a pivotal role in the mechanical hyperalgesia that occurs in OA patients with pain symptoms. Results in models are illustrative: in one rat model, systemic administration of NGF caused mechanical and thermal hyperalgesia [53], while in rat models of OA, intra-articular injection of NGF produced a decrease in the hind paw mechanical withdrawal threshold in one [54] and contributed to spinal nociceptive sensitization in another [55]. Our previous study described a positive correlation between expression levels of NGF mRNA in the synovial membrane and scores for the central sensitization inventory (CSI) and pain in patients with HOA [41]. These findings support previous evidence that monoclonal antibodies against NGF reduce pain symptoms from OA [56-58]. However, evidence for a direct association between PS and CS in human OA and NGF levels in intra-articular tissues such as synovial membrane, synovial fluids, cartilage, etc. is lacking, and further study is required.

Serotonin

In order to modulate spinal nociceptive processing and modulate the descending pain responses, monoaminergic signaling is involved in the process, which originates from the midbrain, the medullary structures, and the subnucleus reticularis dorsalis [59]. Serotonin modulatory effects on pain are complex and dependent on various receptor subtypes being activated. It appears that alterations in serotonergic activity have led to a greater degree of CS. There are several models of persistent neuropathic and inflammatory pain that show upregulated 5-HT receptors in the CNS in models that are driven by pain facilitators such as 5-HT2A receptors [60,61]. It is also known that neuropathic pain models display maladaptive dopaminergic neuroplastic changes, such as a decrease in the expression of D2 receptors in the nucleus accumbens, in addition to these analgesic effects [62]. A variety of persistent pain conditions can be effectively treated with analgesia using drugs that target and improve these monoaminergic systems, such as amitriptyline and serotonin noradrenaline reuptake inhibitors. A higher level of serotonin and dopamine metabolites in the cerebrospinal fluid of OA patients with disabling pain has been associated with increased pain severity and CS [63]. The evidence provided here underlines the fact that CNS monoaminergic activity plays a significant role in the pain processes associated with OA.

Clinical characteristics of sensitized pain in OA

Pain sensitization in people with OA has been assessed using a variety of measures. It is common to perform quantitative sensory testing (QST) as a method of assessment, utilizing standardized mechanical, thermal, or electrical test modalities to assess sensitivity to noxious or innocuous stimuli [64,65]. In a systematic review, PPT data were analyzed in comparison with healthy controls for people with OA. According to the study, pain sensitization was evident at affected and remote anatomical test sites for people with OA [66]. In addition, Lundblad et al. demonstrated that total knee arthroplasty (TKA) for KOA was not always followed by a complete resolution of pain symptoms [67]. Of note, the risk of persistent pain after TKA was increased in subjects with high pre-operative pain scores and low pre-operative local PPTs. In the study using the QST, the purpose was essential to assess the association between the level of the QST and the Western Ontario and McMaster Universities Osteoarthritis Index (WOMAC) post-operative pain after the surgery [68]. The high QST group had more severe WOMAC pain after the surgery at one year compared to the low QST group [68].

In recent years, CS was also assessed by the CSI in patients with OA [69]. This questionnaire, which was designed to evaluate the symptoms associated with CS, includes 25 self-reported items on somatic and emotional symptoms, each of which is scored between 0 and 100 points, with 0 and 100 being the best and worst scores, respectively. According to a 5-point Likert scale, each of the items was graded on a scale of 0 = never, 1 = rarely, 2 = sometimes, 3 = often, and 4 = always. There has been a significant impact on post-operative pain residuals as well as a decrease in satisfaction with CS in studies evaluated by CSI [70-72]. Our previous study suggested that the pre-operative CSI score was negatively correlated with pain and satisfaction scores at 12 months after surgery in patients undergoing total hip arthroplasty (THA) for HOA [70]. Further, a high pre-operative CSI score (>40) is reported to negatively impact post-operative residual pain and satisfaction, as well as the quality of life in patients who underwent TKA for KOA [71,72].

Several studies have described characteristic pain symptoms for detecting sensitized pain. One of these is expanded pain in KOA and HOA. Willett et al. described that expanded pain - assessed by digital pain drawings - was significantly associated with lower PPTs at the thenar eminence, vastus lateralis, and greater trochanter in patients with HOA [73]. Lluch et al. noted that in patients with KOA, the area of expanded pain was associated with lower PPT at the epicondyle and knee and higher CSI scores [74]. Pain at rest is another characteristic of sensitized pain. Satake et al. revealed that the degree of resting pain assessed with a visual analog scale (VAS) was associated with local PPT compared with walking pain in KOA patients [75]. We have reported that VAS resting pain positively correlated with CSI score in patients with HOA [76]. One study suggested that nocturnal pain in KOA is a characteristic symptom of sensitized pain. Sasaki et al. reported that the disability and prevalence of nocturnal pain were higher in KOA patients with CS than in those with non-CS, and found a positive correlation between CSI score and sleep quality determined with the Pittsburgh sleep quality index [77]. These clinical characteristics of sensitized pain are thought to be caused by pathologies of PS and CS.

Treatments for OA pain related to PS and CS

There are several pharmacologic therapies that have the potential to improve OA pain related to PS and CS.

Anti-Inflammatory Cytokine Drugs

Several studies indicated the efficacy and safety of anti-inflammatory cytokine drugs such as human TNF-α or IL-6 monoclonal antibodies for rheumatic diseases, such as RA [78,79]. The evidence on the effectiveness of these drugs for OA pain is limited. A meta-analysis suggested that etanercept and infliximab were superior to placebo for pain in KOA, and infliximab was superior to the other biologic agents (adalimumab, anakinra, canakinumab, etanercept, naproxen, and tocilizumab) in improving pain in the hands and knees of OA [80]. In contrast, several clinical trials have been reported that specifically for OA of the hand, none seem to have shown the efficacy of a monoclonal antibody against TNF-α and IL-6 [81-83]. However, there were no ongoing trials using anti-inflammatory cytokine drugs for OA pain on clinicaltrials.gov.

Tanezumab

Tanezumab is a monoclonal antibody against NGF, which reduces pain symptoms more effectively than other analgesics in moderate-to-severe KOA and HOA [57,84,85]. In a short-term study of KOA and HOA, tanezumab by intravenous administration produced a greater improvement in pain and function than NSAIDs and opioids [85]. A recent phase III randomized controlled trial demonstrated long-term efficacy on subcutaneous administration compared with non-steroidal anti-inflammatory drugs (NSAIDs) in patients with moderate or severe HOA or KOA [84]. However, test group patients were at increased risk of abnormal peripheral sensation and rapidly progressive joint damage compared to the control groups [57,84-86]. NGF inhibitors may effectively improve pain symptoms in OA patients, but the reason why blocking NGF leads to rapid OA progression warrants careful examination.

Duloxetine

Duloxetine, a potent and selective serotonin-norepinephrine reuptake inhibitor, has attracted attention as a potentially useful analgesic for sensitized pain in OA [87]. In RCTs, this agent, which facilitates descending inhibitory pain pathways in the CNS [88], reduced pain and improved function and QOL in patients with KOA and HOA [89-91]. In their 10-week double-blind RCT in patients with severe KOA, Frakes et al. revealed that the addition of duloxetine to oral NSAID therapy offered significant additional pain reduction than NSAIDs alone [92]. Interestingly, pre-operative administration of duloxetine also seems to improve residual pain in the early post-operative period after arthroplasty. Among patients with CS (CSI scores ≥40) and severe KOA, Koh et al. reported that patients receiving duloxetine from the day before surgery to six weeks after surgery had greater pain reduction in the initial 2- to 12-week post-operative period than control patients (no duloxetine) [93]. Future studies should focus on assessing the long-term safety of duloxetine.

Drugs in the ongoing clinical trial phase for OA pain related to PS and CS

There are several drugs with potential future applications for OA pain that are currently in clinical trials (phase II or phase III) (Table 1). This section includes some drugs that target the cannabinoid receptor, TRPV1, and bradykinin B2 receptor in the clinical trial phase registered on www.clinicaltrials.gov for treating OA.

**Table 1:** Drugs in the ongoing clinical trials for osteoarthritis pain related to peripheral and central sensitization

Target	Drug	Mechanism of action	Phase	NCT	Status	Sponsor/Collaborators
Cannabinoid receptors	Cannabidiol	Cannabinoid receptor agonists	Ⅱ	NCT04992624	Recruiting	Richard Harris National Center for Complementary and Integrative Health National Institution Drug Abuse University of Michigan
	Cannabidiol and Cannabinol			NCT04992962	Recruiting	Pure Green
	LY2828360			NCT01319929	Completed	Eli Lilly and Company
TRPV1	RTX-GRT7039	TRPV1 agonist	Ⅲ	NCT05248386	Not yet recruiting	Grünenthal GmbH
				NCT05449132	Not yet recruiting	
				NCT05377489	Not yet recruiting	
	Resiniferatoxin		Ⅱ	NCT04885972	Active, not recruiting	Sorrento Therapeutics, Inc.
Bradykinin B2 receptor	Icatibant	B2 receptor antagonist	Ⅱ	NCT00303056	Completed	Sanofi
	Fasitibant			NCT01091116	Completed	Menarini Group
				NCT02205814	Completed	

Drugs targeting the cannabinoid receptors

The cannabinoid receptors CB1 and CB2 belong to the family of G-protein-coupled receptors and bind exogenous ligands derived from Cannabis sativa as well as endogenous arachidonic-derived ligands. CB2 receptors are primarily expressed in cells of the immune system, including macrophages, and regulate the pro-inflammatory response in various settings [94]. CB2-selective agonists display anti-nociceptive activity in well-validated models of persistent inflammatory pain and neuropathic pain [95]. However, placebo-controlled RCTs indicated that LY2828360, the CB2-selective agonist, lacked both toxicity and efficacy for suppressing KOA pain (clinicaltrials.gov identifier: NCT01319929). Two RCTs in phase II with cannabidiol and cannabinol are currently ongoing for KOA pain (clinicaltrials.gov identifiers: NCT04992624, NCT04992962).

Drugs targeting the TRPV1

The TRP superfamily of ion channels comprises proteins with six transmembrane domains and cytoplasmic N- and C-termini. TRP proteins assemble as homo- or heterotetramers to form cation-permeable ion channels. Twenty-eight TRP channels have been discovered in mammals based on their sequence homology, are classified into six subfamilies [96]. The vanilloid receptor TRPV1 is a homo-tetrameric, non-selective cation channel abundantly expressed in the nociceptors [97]. TRPV1 is considered a validated target for OA pain treatment because its agonists, such as capsaicin, cause desensitization of TRPV1 channels that reduce pain levels in preclinical species, and its antagonists also reduce pain levels in rodent models of OA [98,99].

A recent potential advance in OA pain management is the development of an intra-articular capsaicin formulation, thereby overcoming the likely limited permeability of topical capsaicin into the knee joint [100]. A capsaicin injection into the knee joint was well tolerated and provided dose-dependent improvement in knee OA pain with walking [100]. Currently, two intra-articular injection agents, TRPV1 agonists, are in clinical trials of phases Ⅲ and Ⅱ for patients with KOA (clinicaltrials.gov identifiers: RTX-GRT7039, NCT05248386, NCT05449132, and NCT05377489; Resiniferatoxin, NCT04885972). Further elucidation of the analgesic efficacy and safety of TRPV1 agonists should lead to effective non-opioid analgesic options.

Drugs targeting the bradykinin B2 receptor

Bradykinin is known to have potent pro-inflammatory effects and is one of the most potent endogenous algogenic peptides. This peptide is formed in plasma and inflamed tissues and, by activating the G-protein-coupled receptor, B2 receptor, promotes the activation of nociceptive neurons [44]. Further, elevated bradykinin levels have been demonstrated in the synovial fluid of patients with OA [101]. Thus, bradykinin is an endogenous pro-inflammatory molecule that is associated with the pathophysiology of OA, and B2 receptor antagonists are believed to be considered as a potential symptomatic therapy for this disease. Icatibant and Fasitibant, which are B2 receptor antagonists, have been carried out in phase II of the clinical trials (clinicaltrials.gov identifiers: Icatibant, NCT00303056; Fasitibant, NCT01091116; and NCT02205814). However, no direct evidence of efficacy seems to be indicated. There is a need for further clinical trials to better explain the mechanisms of action and the efficacy and tolerability of the B2 receptor antagonists in OA.

## Conclusions

In this review, we reported findings on the pathophysiology of PS and CS in OA pain and the clinical features and treatment of sensitized pain. Considering the pathophysiology of sensitized pain in OA and the complex clinical features associated with it, accelerating the development of new therapies is important.

Several drugs have been tested in clinical trials to improve sensitized pain caused by OA. Among them, duloxetine appears to be highly efficacious and safe for sensitized pain in OA. Additionally, some drugs targeting cannabinoid receptors, TRPV1 receptors, and bradykinin B2 receptors are currently being tested in clinical trials for the treatment of OA pain caused by PS or CS. These drug targets have the potential to provide better results in alleviating OA pain since they are involved in the pathogenesis of PS and CS. Treatments for sensitized pain in OA are still in their infancy, however, and additional basic and clinical investigations are needed.

## References

[1] Glyn-Jones S, Palmer AJ, Agricola R, Price AJ, Vincent TL, Weinans H, Carr AJ (2015). Osteoarthritis. Lancet.

[2] Martel-Pelletier J, Barr AJ, Cicuttini FM (2016). Osteoarthritis. Nat Rev Dis Primers.

[3] Bedson J, Croft PR (2008). The discordance between clinical and radiographic knee osteoarthritis: a systematic search and summary of the literature. BMC Musculoskelet Disord.

[4] Hattori T, Shimo K, Niwa Y, Tokiwa Y, Matsubara T (2021). Association of chronic pain with radiologic severity and central sensitization in hip osteoarthritis patients. J Pain Res.

[5] López-Ruiz M, Losilla JM, Monfort J (2019). Central sensitization in knee osteoarthritis and fibromyalgia: beyond depression and anxiety. PLoS One.

[6] Ohashi Y, Fukushima K, Uchida K (2022). Differences in outcomes after total hip arthroplasty for osteoarthritis between patients with and without central sensitivity syndromes other than fibromyalgia. Sci Rep.

[7] Thakur M, Dawes JM, McMahon SB (2013). Genomics of pain in osteoarthritis. Osteoarthritis Cartilage.

[8] Schaible HG, Ebersberger A, Natura G (2011). Update on peripheral mechanisms of pain: beyond prostaglandins and cytokines. Arthritis Res Ther.

[9] Lluch E, Torres R, Nijs J, Van Oosterwijck J (2014). Evidence for central sensitization in patients with osteoarthritis pain: a systematic literature review. Eur J Pain.

[10] Lluch Girbés E, Nijs J, Torres-Cueco R, López Cubas C (2013). Pain treatment for patients with osteoarthritis and central sensitization. Phys Ther.

[11] Kidd BL, Urban LA (2001). Mechanisms of inflammatory pain. Br J Anaesth.

[12] Richter F, Natura G, Löser S, Schmidt K, Viisanen H, Schaible HG (2010). Tumor necrosis factor causes persistent sensitization of joint nociceptors to mechanical stimuli in rats. Arthritis Rheum.

[13] Woller SA, Eddinger KA, Corr M, Yaksh TL (2017). An overview of pathways encoding nociception. Clin Exp Rheumatol.

[14] Hunter DJ, McDougall JJ, Keefe FJ (2009). The symptoms of osteoarthritis and the genesis of pain. Med Clin North Am.

[15] Schaible HG, Ebersberger A, Von Banchet GS (2002). Mechanisms of pain in arthritis. Ann N Y Acad Sci.

[16] McDougall JJ, Andruski B, Schuelert N, Hallgrímsson B, Matyas JR (2009). Unravelling the relationship between age, nociception and joint destruction in naturally occurring osteoarthritis of Dunkin Hartley guinea pigs. Pain.

[17] Schuelert N, McDougall JJ (2009). Grading of monosodium iodoacetate-induced osteoarthritis reveals a concentration-dependent sensitization of nociceptors in the knee joint of the rat. Neurosci Lett.

[18] Liu-Bryan R, Terkeltaub R (2015). Emerging regulators of the inflammatory process in osteoarthritis. Nat Rev Rheumatol.

[19] Garland EL (2012). Pain processing in the human nervous system: a selective review of nociceptive and biobehavioral pathways. Prim Care.

[20] Cayrol T, van den Broeke EN (2021). Central sensitisation: causes, therapies, and terminology. Lancet Rheumatol.

[21] Arendt-Nielsen L, Nie H, Laursen MB, Laursen BS, Madeleine P, Simonsen OH, Graven-Nielsen T (2010). Sensitization in patients with painful knee osteoarthritis. Pain.

[22] Bajaj P, Bajaj P, Graven-Nielsen T, Arendt-Nielsen L (2001). Osteoarthritis and its association with muscle hyperalgesia: an experimental controlled study. Pain.

[23] Imamura M, Imamura ST, Kaziyama HH (2008). Impact of nervous system hyperalgesia on pain, disability, and quality of life in patients with knee osteoarthritis: a controlled analysis. Arthritis Rheum.

[24] Mease PJ, Hanna S, Frakes EP, Altman RD (2011). Pain mechanisms in osteoarthritis: understanding the role of central pain and current approaches to its treatment. J Rheumatol.

[25] Fu K, Robbins SR, McDougall JJ (2018). Osteoarthritis: the genesis of pain. Rheumatology (Oxford).

[26] Mathiessen A, Conaghan PG (2017). Synovitis in osteoarthritis: current understanding with therapeutic implications. Arthritis Res Ther.

[27] Wood MJ, Miller RE, Malfait AM (2022). The genesis of pain in osteoarthritis: inflammation as a mediator of osteoarthritis pain. Clin Geriatr Med.

[28] Krustev E, Rioux D, McDougall JJ (2015). Mechanisms and mediators that drive arthritis pain. Curr Osteoporos Rep.

[29] Ren K, Torres R (2009). Role of interleukin-1beta during pain and inflammation. Brain Res Rev.

[30] Manjavachi MN, Motta EM, Marotta DM, Leite DF, Calixto JB (2010). Mechanisms involved in IL-6-induced muscular mechanical hyperalgesia in mice. Pain.

[31] Ghilardi JR, Freeman KT, Jimenez-Andrade JM (2012). Neuroplasticity of sensory and sympathetic nerve fibers in a mouse model of a painful arthritic joint. Arthritis Rheum.

[32] Lee YC, Lu B, Bathon JM, Haythornthwaite JA, Smith MT, Page GG, Edwards RR (2011). Pain sensitivity and pain reactivity in osteoarthritis. Arthritis Care Res (Hoboken).

[33] Leung YY, Huebner JL, Haaland B, Wong SB, Kraus VB (2017). Synovial fluid pro-inflammatory profile differs according to the characteristics of knee pain. Osteoarthritis Cartilage.

[34] Ohashi Y, Uchida K, Fukushima K (2022). Correlation between CD163 expression and resting pain in patients with hip osteoarthritis: possible contribution of CD163+ monocytes/macrophages to pain pathogenesis. J Orthop Res.

[35] Takano S, Uchida K, Inoue G (2017). Nerve growth factor regulation and production by macrophages in osteoarthritic synovium. Clin Exp Immunol.

[36] Yang Z, Lin J, Li H (2022). Bibliometric and visualization analysis of macrophages associated with osteoarthritis from 1991 to 2021. Front Immunol.

[37] Bothwell M (2016). Recent advances in understanding neurotrophin signaling. F1000Res.

[38] Shooter EM (2001). Early days of the nerve growth factor proteins. Annu Rev Neurosci.

[39] Blaney Davidson EN, van Caam AP, Vitters EL (2015). TGF-β is a potent inducer of Nerve Growth Factor in articular cartilage via the ALK5-Smad2/3 pathway. Potential role in OA related pain?. Osteoarthritis Cartilage.

[40] Takano S, Uchida K, Miyagi M (2016). Nerve growth factor regulation by TNF-α and IL-1β in synovial macrophages and fibroblasts in osteoarthritic mice. J Immunol Res.

[41] Ohashi Y, Uchida K, Fukushima K (2021). NGF expression and elevation in hip osteoarthritis patients with pain and central sensitization. Biomed Res Int.

[42] Mantyh PW, Koltzenburg M, Mendell LM, Tive L, Shelton DL (2011). Antagonism of nerve growth factor-TrkA signaling and the relief of pain. Anesthesiology.

[43] Averill S, McMahon SB, Clary DO, Reichardt LF, Priestley JV (1995). Immunocytochemical localization of trkA receptors in chemically identified subgroups of adult rat sensory neurons. Eur J Neurosci.

[44] Lee YJ, Zachrisson O, Tonge DA, McNaughton PA (2002). Upregulation of bradykinin B2 receptor expression by neurotrophic factors and nerve injury in mouse sensory neurons. Mol Cell Neurosci.

[45] Lesser SS, Lo DC (1995). Regulation of voltage-gated ion channels by NGF and ciliary neurotrophic factor in SK-N-SH neuroblastoma cells. J Neurosci.

[46] Mamet J, Baron A, Lazdunski M, Voilley N (2002). Proinflammatory mediators, stimulators of sensory neuron excitability via the expression of acid-sensing ion channels. J Neurosci.

[47] Zhang X, Huang J, McNaughton PA (2005). NGF rapidly increases membrane expression of TRPV1 heat-gated ion channels. EMBO J.

[48] Levine ES, Dreyfus CF, Black IB, Plummer MR (1995). Differential effects of NGF and BDNF on voltage-gated calcium currents in embryonic basal forebrain neurons. J Neurosci.

[49] Michael GJ, Averill S, Nitkunan A, Rattray M, Bennett DL, Yan Q, Priestley JV (1997). Nerve growth factor treatment increases brain-derived neurotrophic factor selectively in TrkA-expressing dorsal root ganglion cells and in their central terminations within the spinal cord. J Neurosci.

[50] Park KA, Fehrenbacher JC, Thompson EL, Duarte DB, Hingtgen CM, Vasko MR (2010). Signaling pathways that mediate nerve growth factor-induced increase in expression and release of calcitonin gene-related peptide from sensory neurons. Neuroscience.

[51] Skoff AM, Adler JE (2006). Nerve growth factor regulates substance P in adult sensory neurons through both TrkA and p75 receptors. Exp Neurol.

[52] Caumo W, Deitos A, Carvalho S (2016). Motor cortex excitability and BDNF levels in chronic musculoskeletal pain according to structural pathology. Front Hum Neurosci.

[53] Lewin GR, Rueff A, Mendell LM (1994). Peripheral and central mechanisms of NGF-induced hyperalgesia. Eur J Neurosci.

[54] Ashraf S, Mapp PI, Burston J, Bennett AJ, Chapman V, Walsh DA (2014). Augmented pain behavioural responses to intra-articular injection of nerve growth factor in two animal models of osteoarthritis. Ann Rheum Dis.

[55] Sagar DR, Nwosu L, Walsh DA, Chapman V (2015). Dissecting the contribution of knee joint NGF to spinal nociceptive sensitization in a model of OA pain in the rat. Osteoarthritis Cartilage.

[56] Bannwarth B, Kostine M (2017). Nerve growth factor antagonists: is the future of monoclonal antibodies becoming clearer?. Drugs.

[57] Schmelz M, Mantyh P, Malfait AM, Farrar J, Yaksh T, Tive L, Viktrup L (2019). Nerve growth factor antibody for the treatment of osteoarthritis pain and chronic low-back pain: mechanism of action in the context of efficacy and safety. Pain.

[58] Wise BL, Seidel MF, Lane NE (2021). The evolution of nerve growth factor inhibition in clinical medicine. Nat Rev Rheumatol.

[59] Ossipov MH, Dussor GO, Porreca F (2010). Central modulation of pain. J Clin Invest.

[60] Jeong HJ, Mitchell VA, Vaughan CW (2012). Role of 5-HT(1) receptor subtypes in the modulation of pain and synaptic transmission in rat spinal superficial dorsal horn. Br J Pharmacol.

[61] Liu QQ, Yao XX, Gao SH, Li R, Li BJ, Yang W, Cui RJ (2020). Role of 5-HT receptors in neuropathic pain: potential therapeutic implications. Pharmacol Res.

[62] Kishikawa Y, Kawahara Y, Ohnishi YN, Sotogaku N, Koeda T, Kawahara H, Nishi A (2022). Dysregulation of dopamine neurotransmission in the nucleus accumbens in immobilization-induced hypersensitivity. Front Pharmacol.

[63] Bjurström MF, Blennow K, Zetterberg H (2022). Central nervous system monoaminergic activity in hip osteoarthritis patients with disabling pain: associations with pain severity and central sensitization. Pain Rep.

[64] Pavlaković G, Petzke F (2010). The role of quantitative sensory testing in the evaluation of musculoskeletal pain conditions. Curr Rheumatol Rep.

[65] Rudy-Froese B, Rankin J, Hoyt C, Ramsahoi K, Gareau L, Howatt W, Carlesso LC (2021). Quantitative sensory testing protocols to evaluate central and peripheral sensitization in knee OA: a protocol for a scoping review. Curr Rheumatol Rev.

[66] Fingleton C, Smart K, Moloney N, Fullen BM, Doody C (2015). Pain sensitization in people with knee osteoarthritis: a systematic review and meta-analysis. Osteoarthritis Cartilage.

[67] Lundblad H, Kreicbergs A, Jansson KA (2008). Prediction of persistent pain after total knee replacement for osteoarthritis. J Bone Joint Surg Br.

[68] Wylde V, Palmer S, Learmonth ID, Dieppe P (2013). The association between pre-operative pain sensitisation and chronic pain after knee replacement: an exploratory study. Osteoarthritis Cartilage.

[69] Roby NU, Packham TL, MacDermid JC, Carlesso LC (2022). Validity of the Central Sensitization Inventory (CSI) through Rasch analysis in patients with knee osteoarthritis. Clin Rheumatol.

[70] Ohashi Y, Fukushima K, Uchida K (2021). Adverse effects of higher preoperative pain at rest, a central sensitization-related symptom, on outcomes after total hip arthroplasty in patients with osteoarthritis. J Pain Res.

[71] Koh IJ, Kang BM, Kim MS, Choi KY, Sohn S, In Y (2020). How does preoperative central sensitization affect quality of life following total knee arthroplasty?. J Arthroplasty.

[72] Kim SH, Yoon KB, Yoon DM, Yoo JH, Ahn KR (2015). Influence of centrally mediated symptoms on postoperative pain in osteoarthritis patients undergoing total knee arthroplasty: a prospective observational evaluation. Pain Pract.

[73] Willett MJ, Siebertz M, Petzke F (2020). The extent of pain is associated with signs of central sensitization in patients with hip osteoarthritis. Pain Pract.

[74] Lluch Girbés E, Dueñas L, Barbero M (2016). Expanded distribution of pain as a sign of central sensitization in individuals with symptomatic knee osteoarthritis. Phys Ther.

[75] Satake Y, Izumi M, Aso K, Igarashi Y, Sasaki N, Ikeuchi M (2021). Comparison of predisposing factors between pain on walking and pain at rest in patients with knee osteoarthritis. J Pain Res.

[76] Ohashi Y, Fukushima K, Inoue G (2020). Central sensitization inventory scores correlate with pain at rest in patients with hip osteoarthritis: a retrospective study. BMC Musculoskelet Disord.

[77] Sasaki E, Ota S, Chiba D (2021). Association between central sensitization and increasing prevalence of nocturnal knee pain in the general population with osteoarthritis from the Iwaki cohort study. J Pain Res.

[78] Bijlsma JWJ, Welsing PMJ, Woodworth TG (2016). Early rheumatoid arthritis treated with tocilizumab, methotrexate, or their combination (U-Act-Early): a multicentre, randomised, double-blind, double-dummy, strategy trial. Lancet.

[79] Smolen JS, Emery P, Fleischmann R (2014). Adjustment of therapy in rheumatoid arthritis on the basis of achievement of stable low disease activity with adalimumab plus methotrexate or methotrexate alone: the randomised controlled OPTIMA trial. Lancet.

[80] Li Y, Mai Y, Cao P (2022). Relative efficacy and safety of anti-inflammatory biologic agents for osteoarthritis: a conventional and network meta-analysis. J Clin Med.

[81] Chevalier X, Ravaud P, Maheu E (2015). Adalimumab in patients with hand osteoarthritis refractory to analgesics and NSAIDs: a randomised, multicentre, double-blind, placebo-controlled trial. Ann Rheum Dis.

[82] Richette P, Latourte A, Sellam J (2021). Efficacy of tocilizumab in patients with hand osteoarthritis: double blind, randomised, placebo-controlled, multicentre trial. Ann Rheum Dis.

[83] Aitken D, Laslett LL, Pan F (2018). A randomised double-blind placebo-controlled crossover trial of HUMira (adalimumab) for erosive hand OsteoaRthritis - the HUMOR trial. Osteoarthritis Cartilage.

[84] Hochberg MC, Carrino JA, Schnitzer TJ (2021). Long-term safety and efficacy of subcutaneous tanezumab versus nonsteroidal antiinflammatory drugs for hip or knee osteoarthritis: a randomized trial. Arthritis Rheumatol.

[85] Schnitzer TJ, Marks JA (2015). A systematic review of the efficacy and general safety of antibodies to NGF in the treatment of OA of the hip or knee. Osteoarthritis Cartilage.

[86] Neogi T, Hunter DJ, Churchill M (2022). Observed efficacy and clinically important improvements in participants with osteoarthritis treated with subcutaneous tanezumab: results from a 56-week randomized NSAID-controlled study. Arthritis Res Ther.

[87] Weng C, Xu J, Wang Q, Lu W, Liu Z (2020). Efficacy and safety of duloxetine in osteoarthritis or chronic low back pain: a Systematic review and meta-analysis. Osteoarthritis Cartilage.

[88] Woolf CJ (2004). Pain: moving from symptom control toward mechanism-specific pharmacologic management. Ann Intern Med.

[89] Blikman T, Rienstra W, van Raaij TM (2022). Duloxetine in OsteoArthritis (DOA) study: effects of duloxetine on pain and function in end-stage hip and knee OA - a pragmatic enriched randomized controlled trial. BMC Musculoskelet Disord.

[90] Enomoto H, Fujikoshi S, Ogawa K, Tsuji T, Tanaka S (2020). Relationship between pain reduction and improvement in health-related quality of life in patients with knee pain due to osteoarthritis receiving duloxetine: exploratory post hoc analysis of a Japanese phase 3 randomized study. J Pain Res.

[91] Enomoto H, Fujikoshi S, Tsuji T, Sasaki N, Tokuoka H, Uchio Y (2018). Efficacy of duloxetine by prior NSAID use in the treatment of chronic osteoarthritis knee pain: A post hoc subgroup analysis of a randomized, placebo-controlled, phase 3 study in Japan. J Orthop Sci.

[92] Frakes EP, Risser RC, Ball TD, Hochberg MC, Wohlreich MM (2011). Duloxetine added to oral nonsteroidal anti-inflammatory drugs for treatment of knee pain due to osteoarthritis: results of a randomized, double-blind, placebo-controlled trial. Curr Med Res Opin.

[93] Koh IJ, Kim MS, Sohn S, Song KY, Choi NY, In Y (2019). Duloxetine reduces pain and improves quality of recovery following total knee arthroplasty in centrally sensitized patients: a prospective, randomized controlled study. J Bone Joint Surg Am.

[94] Turcotte C, Blanchet MR, Laviolette M, Flamand N (2016). The CB(2) receptor and its role as a regulator of inflammation. Cell Mol Life Sci.

[95] Guindon J, Hohmann AG (2008). Cannabinoid CB2 receptors: a therapeutic target for the treatment of inflammatory and neuropathic pain. Br J Pharmacol.

[96] Samanta A, Hughes TE, Moiseenkova-Bell VY (2018). Transient receptor potential (TRP) channels. Subcell Biochem.

[97] Wong GY, Gavva NR (2009). Therapeutic potential of vanilloid receptor TRPV1 agonists and antagonists as analgesics: recent advances and setbacks. Brain Res Rev.

[98] Chu KL, Chandran P, Joshi SK, Jarvis MF, Kym PR, McGaraughty S (2011). TRPV1-related modulation of spinal neuronal activity and behavior in a rat model of osteoarthritic pain. Brain Res.

[99] Honore P, Chandran P, Hernandez G (2009). Repeated dosing of ABT-102, a potent and selective TRPV1 antagonist, enhances TRPV1-mediated analgesic activity in rodents, but attenuates antagonist-induced hyperthermia. Pain.

[100] Stevens RM, Ervin J, Nezzer J (2019). Randomized, double-blind, placebo-controlled trial of intraarticular trans-capsaicin for pain associated with osteoarthritis of the knee. Arthritis Rheumatol.

[101] Bellucci F, Meini S, Cucchi P (2013). Synovial fluid levels of bradykinin correlate with biochemical markers for cartilage degradation and inflammation in knee osteoarthritis. Osteoarthritis Cartilage.

